# Pathogenicity and Cytokine Gene Expression Pattern of a Serotype 4 Fowl Adenovirus Isolate

**DOI:** 10.1371/journal.pone.0077601

**Published:** 2013-10-18

**Authors:** Helena Grgić, Zvonimir Poljak, Shayan Sharif, Éva Nagy

**Affiliations:** 1 Department of Pathobiology, Ontario Veterinary College, University of Guelph, Guelph, Ontario, Canada; 2 Department of Population Medicine, Ontario Veterinary College, University of Guelph, Guelph, Ontario, Canada; French National Centre for Scientific Research, France

## Abstract

Hydropericardium-hepatitis syndrome (HHS), a recently emerged disease of chickens, is caused by some strains of fowl adenovirus serotype 4 (FAdV-4). In this study, a Canadian FAdV-4 isolate, designated as FAdV-4 ON1, was evaluated for pathogenicity after oral and intramuscular (im) infection of specific pathogen free (SPF) chickens. Pathogenicity was evaluated by observation of clinical signs and gross and histological lesions. The highest viral DNA copy numbers, irrespective of the inoculation route, were detected in the cecal tonsils. Virus titers in cloacal swabs collected over the entire study period were compared between the orally and im inoculated chickens, and the difference in titers between the two groups was significant (P<0.001), the oral group had a higher rank. The antibody response of infected chickens tested by an adenovirus-specific ELISA showed a statistically significant (P<0.001) difference between the orally and im inoculated chickens. The im inoculated chickens had higher values than birds inoculated orally (P<0.001). Serum samples from both groups collected at 14 days post-infection completely neutralized FAdV-4 ON1. In addition, the effects of FAdV-4 ON1 infection on transcription of a number of avian cytokines were studied *in vivo*. The expression of interferon (IFN)-γ and interleukin (IL)-10 in the liver was induced at early times after infection. This FAdV-4 ON1 potentially could be used as a live vaccine against HHS and developed as vaccine vector.

The GenBank/EMBL/DDBJ accession number for the FAdV-4 ON1 sequence is GU188428.

##  Introduction

Fowl adenovirus serotype 4 (FAdV-4) plays a major role in the aetiology of a very important disease in broiler chickens called hydropericardium-hepatitis syndrome (HHS). It was first seen in Pakistan in 1987 [23] and since then outbreaks have also been reported in India, the Middle East, Japan, Mexico, Peru, Ecuador, and Chile [3,10,20,40] causing significant losses to the poultry industry. However, no HHS has been reported in Canada. The disease is prevalent in 3- to 6-week-old broiler chickens. The affected birds may not exhibit clinical signs other than high mortality, up to 75% [18]. The predominant gross lesion is hydropericardium, characterized by accumulation of a gelatinous material in the pericardial sac. The heart appears misshapen and flabby, with its apex floating in the pericardial sac [4,26]. At histological examination, the most consistent findings in the liver are small multifocal areas of necrosis and mononuclear cell infiltration, including basophilic intranuclear inclusion bodies in hepatocytes surrounded by a clear halo or filling the entire nucleus [27]. HHS associated losses could be reduced by vaccination. Inactivated liver homogenates from infected chickens, and inactivated virus propagated in cell culture and/or embryonated eggs based vaccines are available, however they provide only variable protection [6,25]. Recently, Schonewille et al. (2010) reported live FAdV-4 vaccine, but with lack of neutralizing antibodies. 

 Although there is no history of HHS in Canada, in 2004 a serotype 4 fowl adenovirus was isolated for the first time from a broiler breeder flock with no clinical signs of HHS. The complete nucleotide sequence of this virus (FAdV-4 ON1) was determined by Griffin and Nagy [18]. FAdVs are excellent vaccine vectors, and both FAdV-8 [38,22] and FAdV-9 [7,8,28] have been developed as vectors. This newly identified FAdV-4 ON1 could be considered not only as a live vaccine virus against HHS but as a vector virus as well. 

The goals of this study were to 1) determine the pathogenicity of the FAdV-4 ON1 isolate in specific pathogen free (SPF) chickens, 2) investigate virus dissemination in tissues, virus titers in cloacal swabs, and antibody response, and 3) examine the dynamics of interferon (IFN)-γ, interleukin (IL)-10, IL-18, and IL-8 gene expression after FAdV-4 ON1 infection.

## Materials and Methods

### Virus

 The FAdV-4 was isolated in 2004 by the Animal Health Laboratory at the University of Guelph. The sample came from a Canadian broiler breeder flock showing no clinical signs of HHS. The virus was plaque purified, and the designated virus (FAdV-4 ON1) was propagated and titrated in a continuous chicken hepatoma cell line (CH-SAH, Solvay Animal Health, Mendota Heights, Minnesota, USA ) as described [5]. The complete genome sequence of the virus was determined [18] and the GenBank/EMBL/DDBJ accession number is GU188428.

### Chickens and housing

White Leghorn SPF chicken eggs were obtained from the Canadian Food Inspection Agency, Ottawa, and they were hatched at the Arkell Poultry Station of the University of Guelph. The birds were housed at the Isolation Unit according to the Animal Care Committee of the University of the Guelph in accordance with the *Guide to the Care and Use of Experimental Animals of The Canadian Council on Animal Care.*

(http://www.uoguelph.ca/research/assets/acs/docs/university_animal_care_policy_and_procedures.pdf)

### Pathogenicity assessment

A trial was conducted to evaluate the pathogenicity of the FAdV-4 ON1 isolate according to the University of Guelph Animal Care Committee regulations. The protocol was approved by the University of Guelph Animal Care Committee (Animal Utilization Protocol number 08R126). One hundred chicks were randomly divided into four groups: Group I (FAdV-4) -oral, Group II (FAdV-4) -intramuscular (im), Group III (Control) -oral, and Group IV (Control) -im. Groups I and II were composed of 30 chicks each, and the negative control groups (III and IV) were composed of 20 chicks each. The chicks were tagged with wing bands at 9 days of age and blood samples were collected. Since HHS is not reported in newly hatched birds, ten-day-old chickens in Groups I and II were inoculated with 2x10^8^ plaque forming units (pfu) of virus per chick. The control chicks (mock-infected) received phosphate buffered saline (PBS). Groups I and II were re-inoculated at 14 days of age with the same dose of virus, in order to provide a sufficiently high dose of the virus that could induce clinical signs and to mimic the continuous infection that occurs in a barn. The chicks were observed daily for clinical signs. Three chicks from the control and four chicks from the treatment groups were randomly selected for necropsy at 0, 3, 5, 7, 14, 21, and 28 days post-infection (d.p.i.). The chickens were euthanized with carbon dioxide gas according to the University of Guelph Animal Care Committee regulations, necropsied, and examined for the presence of gross lesions. Tissues from thymus, lung, heart, kidney, liver, bursa of Fabricius, and cecal tonsils were collected. Each organ was sectioned into two portions: one part was placed into 10% formalin, and the other one was stored at -80°C for quantitative polymerase chain reaction (qPCR). Cloacal swabs were collected at 0, 3, 5, 10, 14, 21, 28 d.p.i. and their virus titers were determined by a plaque assay. Blood samples were collected and tested by ELISA and virus neutralization test.

### Serologic testing

The serum samples collected at 0, 7, 14, 21, and 28 d.p.i. were tested for FAdV-specific antibodies (Abs) by ELISA as described [29]. Briefly, microtiter plates (Becton-Dickinson, Franklin Lakes, New Jersey, USA) were coated with FAdV-4 antigen treated with *N*-lauroyl sarcosine at a concentration of 200 ng/well and blocked with 3% bovine serum albumin. The bound antibody was detected by alkaline-phosphatase-labeled goat IgG conjugate against chicken immunoglobulin (Kirkegaard and Perry Laboratories, Gaithersburg, Maryland, USA) and Sigma Fast *p*-nitrophenyl phosphate (Sigma Chemical Company, St. Louis, Missouri, USA).

The results were expressed by calculating the sample-to-positive (S/P) ratio for each sample from mean optical density (OD_405_) readings. 

 The serum samples were also assayed for virus neutralizing antibodies [32]. Briefly, 100 pfu of the virus in 100 μl of cell culture medium were mixed with equal volume of 1:50 dilutions of Abs. The mixture was incubated for 1 h, and the sample was then added to CH-SAH cells. After a 1 h adsorption period, the cells were overlaid with a 0.6% agarose. The plaques were visualized by addition of a 2nd overlay after 72 h of incubation at 37°C in 5% CO_2_ containing 0.02% neutral red. A sample that inhibited the development of more than 50% of the plaques compared with the negative-control serum sample was considered positive.

### Experimental design for determination of cytokine mRNA expression

Forty ten-day-old chicks were randomly divided into two groups. Group I was composed of 25 birds and Group II (negative control) contained 15 birds. Each bird in Group I received 2x10^8^ pfu/ml of the virus im and the control chicks received PBS. Five chicks from the infected and three chicks from the control group were selected randomly for necropsy at 1, 3, 5, 7, and 10 d.p.i. The rationale for selecting these time points was the assumption that one immune measure at one time point is rarely sufficient. Moreover, FAdV infection results in viraemia by 24 hours p.i. with two peaks, the first at day 2 and the second at day 7 p.i. [36]. The chicks were euthanized and the spleen, cecal tonsil, and liver were collected and submerged in RNAlater (Qiagen, Mississauga, ON, Canada) and stored at -80°C until RNA extraction. 

### DNA extraction and quantification of viral DNA in tissues

Total DNA was extracted with DNeasy^®^ tissue kit (QIAGEN InC., Mississauga) and the viral load in the tissues was determined by qPCR with SYBR Green as an intercalating dye. The FAdV-4 ON1 ORF14 gene was used as an indicator for the presence of viral DNA. The forward and reverse primers were 5’-AGTGTGTATGTGCGTTGGGTAG-3’ and 5’-CATTGTCATAACGATGGTGTAG-3’, respectively. The qPCR reaction was carried out as described [16].

### RNA extraction and real-time quantitative PCR for determination of cytokine gene expression

Total RNA was extracted from tissues by Trizol reagent (Invitrogen, Canada Inc., Burlington, ON, Canada) as described by the manufacturer. The tissues were homogenized in 1 ml Trizol followed by chloroform extraction, isopropanol precipitation, and a 75% ethanol wash. The RNA pellet was dissolved in 20 µl of RNAse-free water. The concentration of the RNA was measured by a NanoVue spectrophotometer. cDNA was synthesized from total RNA with SuperScript^TM^ II Reverse Trancriptase (Invitrogen) according to the manufacturer’s instructions, and the random primers were from Invitrogen. The cDNAs were stored at -80°C until use.

A real-time quantitative PCR (RT-qPCR) assay was employed to quantify the expression of β-actin and cytokine genes in cDNA samples. The reaction was prepared in a total volume of 20 µl of Qiagen Quantitect SYBR Green PCR kit (Qiagen) containing hot start Taq polymerase and cDNA. Each assay was performed with a no-template control and in triplicates of the standard in a LightCycler (LC480) instrument (Roche Diagnostics GmbH, Mannheim, Germany).

 The sequences and other features of specific primers for chicken IFN-γ, IL-18, IL-10, IL-8, and β-actin have been described [1,2] and they are given in Table 1. The primers were synthesized by Laboratory Services, University of Guelph. The PCR conditions for different segments of each cycle were optimized for all target genes.

**Table 1:** The sequences of the primers utilized in study.

**Target**	**Sequences**
**IL-18**	Forward 5'-GAAACGTCAATAGCCAGTTGC-3'
	Reverse 5'-TCCCATGCTCTTTCTCACAACA-3'
**IL-10**	Forward 5'-AGCAGATCAAGGAGACGTTC-3'
	Reverse 5'-ATCAGCAGGTACTCCTCGAT-3'
**IL-8**	Forward 5’-TATCACTGGAGAGTCTCGCTGTC-3’
	Reverse 5’-TCAGTCCTCCTGCAGTGTATACC-3’
**IFN-γ**	Forward 5' - ACACTGACAAGTAAAGCCGCACA-3'
	Reverse 5' -AGTCGTTCATCGGGAGCTTGGC-3'
**β-actin**	Forward 5'-CAACACAGTGCTGTCTGGTGGTA-3'
	Reverse 5'-ATCGTACTCCTGCTTGCTGATCC-3'

Specific primers for chicken IFN-γ, IL-18, IL-8 were described by Abdul-Careem et al [1], and for IL-10, and β-actin were described by Abdul-Careem et al [2].

Since the most variability in cytokine gene expression following FAdV-8 infection was in the liver [15], mRNA expression in liver was determined for IFN-γ, IL-18, IL-10, and IL-8, while for cecal tonsil and spleen, mRNA expression was determined only for IFN-γ and IL-18.

### Statistical analysis

To evaluate if viral copy number differed among tissues, a Kruskal-Wallis test was performed. Data from the oral and im groups were subjected separately to the Kruskal-Wallis test to evaluate the differences in number of viral copies among tissues. When statistically significant *P*-values were identified, pair-wise comparisons were performed between different tissues using the Mann-Whitney U test. *P*-values of <0.05 were considered to be statistically significant. 

Real-time quantitative PCR efficiency was calculated as described [35], and mRNA expression was calculated relative to β-actin gene expression [31]. The difference in cytokine expression between groups was assessed by a Mann-Whitney U test and comparisons were considered significant at *P*<0.05.

## Results

### Clinical signs, gross pathology and histology

Clinical signs indicative of HHS and characterized by depression, chickens huddling in corners with ruffled feathers, and chickens exhibiting the characteristic posture, with the chest and beak resting on the ground, were not seen in any chicks. Distinctive gross lesions, hydropericardium, and pale, swollen and friable liver were absent. The characteristic histological signs of HHS described as intranuclear inclusion bodies in hepatocytes were also not detected in any of the infected chickens. 

### Viral genome copy number in tissues

Viral genome copy numbers in liver, bursa of Fabricius, and cecal tonsil were determined by qPCR and the results are summarized in Table 2. No viral DNA was detected in any tissues from any chickens before inoculation or in the mock-infected chickens. The cecal tonsil was the organ with the highest number of viral copies, followed by the liver and then the bursa of Fabricius, irrespective of the inoculation route. For the orally inoculated birds, viral copy number was higher in cecal tonsil than in liver (P=0.0207), and bursa of Fabricius (P=0.0001), whereas viral copy numbers in the liver ranked only marginally higher than viral copy numbers in the bursa of Fabricius (P=0.0759). For the im inoculated birds, viral copy number was higher in cecal tonsil than in the bursa (P=0.0154), whereas differences among other organs were not significant (P>0.25). 

**Table 2:** Number of positive tissues and viral genome copy numbers in tissues of chickens inoculated with FAdV-4.

**Days p.i.**		**Inoculation route**				
	**Tissue**	**Oral**			**Intramuscular**	
	Liver	3.4^a^	3/4^b^		7.5	2/4
3	Bursa	5.6	2/4		5	1/4
	Cecal tonsil	26	4/4		530	4/4
	Liver	140	3/4		8.3	4/4
5	Bursa	6.1	4/4		12	2/4
	Cecal tonsil	570	4/4		130	4/4
	Liver	2.2	2/4		2.3	2/4
7	Bursa	0	0/4		6	2/4
	Cecal tonsil	24	1/4		3.5	3/4
	Liver	2.8	4/4		5.9	2/4
14	Bursa	4.9	3/4		4.6	2/4
	Cecal tonsil	12	4/4		8.6	4/4
	Liver	11	4/4		24	4/4
21	Bursa	23	3/4		310	4/4
	Cecal tonsil	6400	4/4		360	3/4
	Liver	650	4/4		350	4/4
28	Bursa	1.4	2/4		74	1/4
	Cecal tonsil	170	4/4		650	4/4

a = viral genome copy number/10 µg of total DNA determined by qPCR. b = number of positive tissues/number analyzed tissues

### Virus shedding

 No virus was detected in the cloacal swabs of any chickens before inoculation or in the mock-infected groups at any time (data not shown). The virus titers in the cloacal swabs of chickens inoculated with FAdV-4 ON1 are given in Table 3. 

**Table 3:** Virus titers in the cloacal swabs of chickens inoculated with FAdV-4.

**Days**		**Inoculation route**		
**post-infection**		**Mean titers (pfu/ml**)** in swabs**		
		**Chickens shedding the virus (%)^a^**		
		**Oral**	**Intramuscular**	***P*-value**
3		9.9x10^3^	1.4x10^3^	≤0.001
		(93)	(25)	
5		1.2x10^4^	1.2x10^3^	≤0.001
		(83)	(32)	
10		1.3x10^2^	6.8x10^2^	0.49
		(13)	(20)	
14		3.9x10^1^	2.5x10^1^	0.26
		(27)	(13)	
21		3.3x10^1^	2.5x10^1^	0.25
		(20)	(10)	
28		0	1.2x10^1^	0.34
		(0)	(10)	

The virus titers were determined by plaque assay.

P values < 0.05 are statistically significant for comparison between oral and im on each day.

a 26 and 22 chickens at days 3 and 5, respectively; 18 and 14 chickens at days 10 and 14, respectively; 10 and 6 chickens at days 21 and 28, respectively.

 The difference in titers between the groups of orally and im inoculated chickens was statistically significant (P<0.001) at 3 and 5 d.p.i., when tested by the Mann-Whitney U test. The oral group had higher ranks. Chickens in the im group shed the virus throughout the experiment (28 d.p.i), while virus shedding in the oral group was recorded until 21 d.p.i. .

### Antibody response

**Figure 1:**
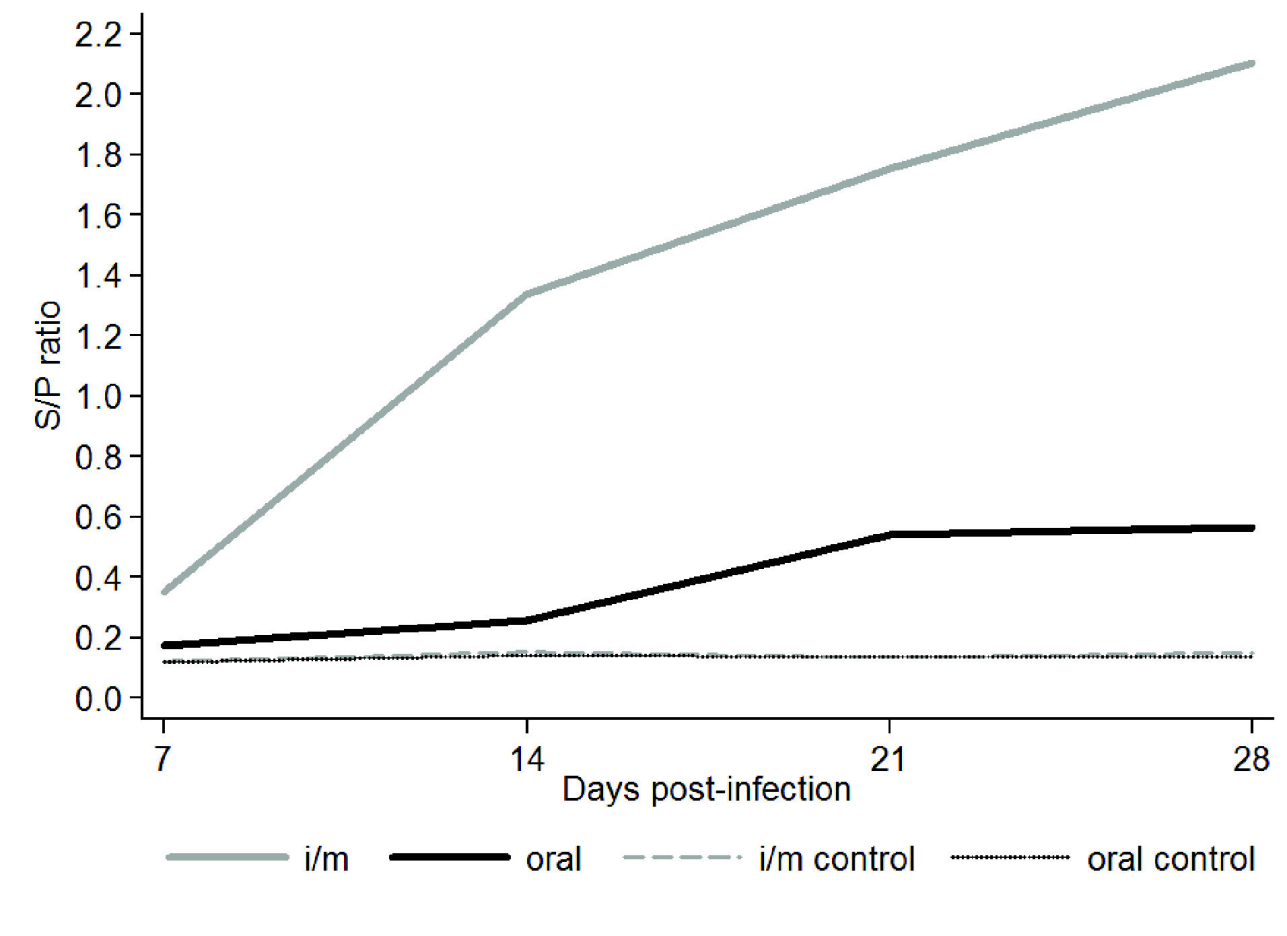
Antibody response to viral proteins in chickens inoculated with FAdV-4 by the oral and intramuscular routes and mock infected as measured by S/P ratios. Serum samples were collected at 7, 14, 21 and 28 days post-infection. Gray and black lines represent im and orally inoculated chickens, respectively. Dashed gray line and black dotted line represent mock-infected chickens. Statisticaly significant differences among mock infected and infected birds have been detected at 14, 21, and 28 d.p.i.

 FAdV specific Ab was not detected in any birds before inoculation nor in the mock-infected birds. The Ab response against FAdV-4, shown in Figure 1, appeared at 7 d.p.i. and the difference between inoculated groups and negative controls was statistically significant (P<0.001) at 14, 21 and 28 d.p.i. The im inoculated chickens had higher values than birds inoculated orally (P<0.001). When the same sera were tested against another serotype, FAdV-9, the results were similar, but the Ab levels were slightly lower (data not shown). 

### Virus neutralization assay

 No neutralizing antibodies were found in any birds prior to the challenge. 

In the virus neutralization test, all serum samples collected from both the oral and im groups after 14 days of infection completely neutralized the FAdV-4 ON1 in 1:50 dilution. Finally, negative control birds were negative throughout the whole study.

### Expression of cytokine genes

 Expression of mRNA of cytokine genes after im inoculation of chickens with FAdV-4 ON1 was measured in the cecal tonsil, liver, and spleen. 

**Figure 2:**
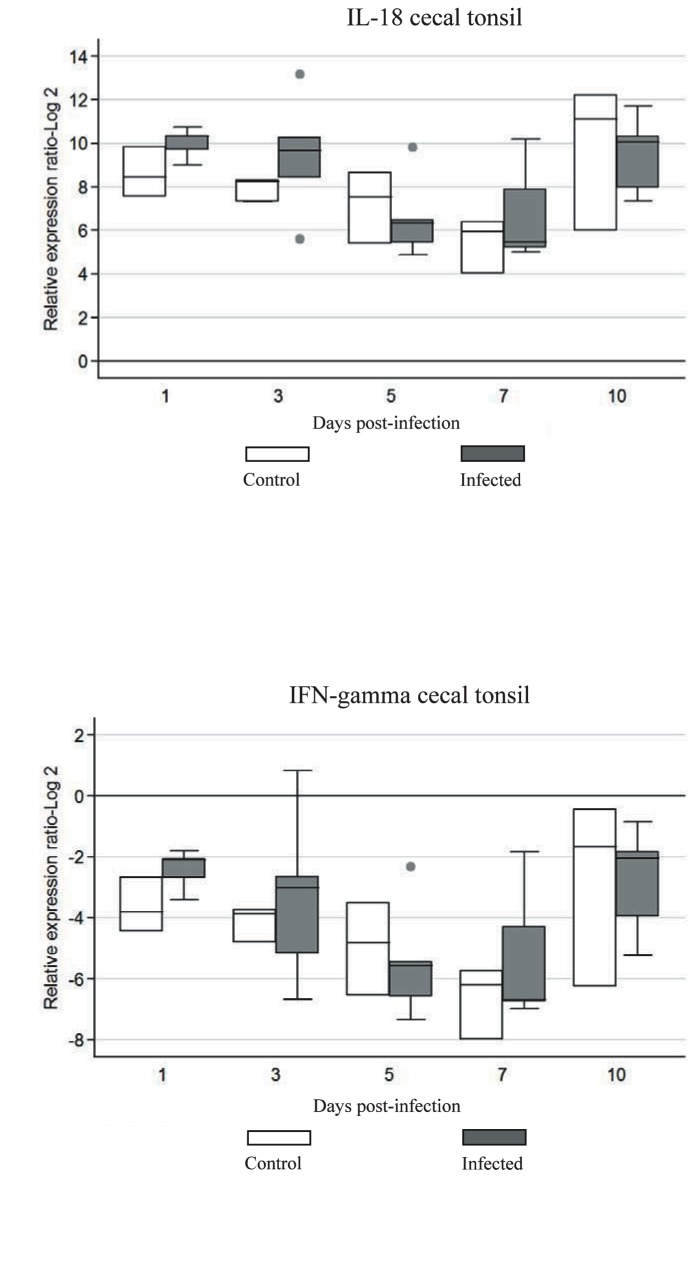
Cytokine mRNA expression in cecal tonsil of chickens infected with FAdV-4. The groups were: uninfected (negative control), and FAdV-4 infected chickens using im inoculation. Target and reference gene expression was quantified by real-time RT-PCR using SYBR Green. Target gene expression is presented relative to β-actin expression and normalized to a calibrator. The results are diagrammed as whisker boxes with medians. Boxes represent interquartile ranges and whiskers indicate extreme values. There was no statistically significant differences detected (P>0.05) by Mann-Whitney test. ● symbols represent values which were identified as outliers.

In the cecal tonsils, there were no significant differences (*P*>0.05) in the expression of IFN-γ and IL-18 mRNA when compared with the values for the uninfected controls (Figure 2). 

**Figure 3:**
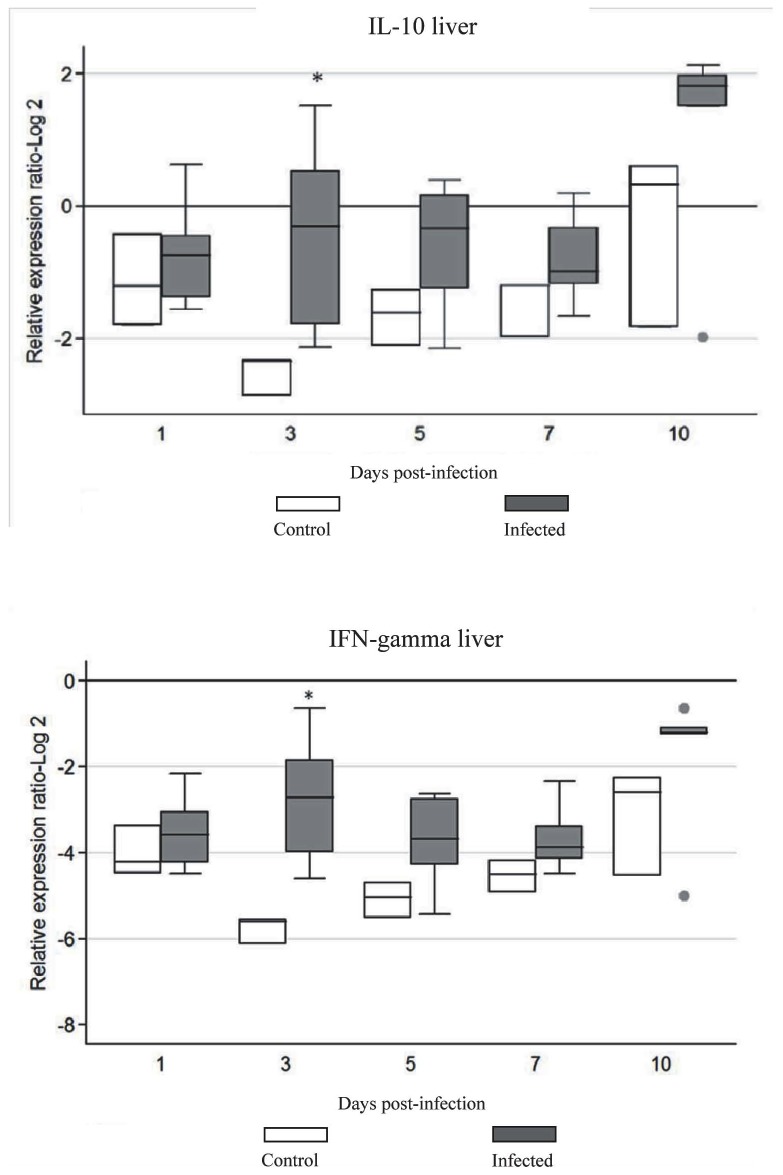
Cytokine mRNA expression in liver of chickens infected with FAdV-4. The groups were: uninfected (negative control), and FAdV-4 infected chickens using im inoculation. Target and reference gene expression was quantified by real-time RT-PCR using SYBR Green. Target gene expression is presented relative to β-actin expression and normalized to a calibrator. The results are diagrammed as whisker boxes with medians. Boxes represent interquartile ranges and whiskers indicate extreme values. The difference in cytokine expression between groups was assessed by Mann-Whitney test and comparisons were considered significant at P≤0.05 (*).● symbols represent values which were identified as outliers.

The expression of IFN-γ, IL-18, IL-10, and IL-8 mRNA is illustrated in Figure 3. There was a statistically significant increase (*P*<0.05) in IFN-γ gene expression of infected chickens at 3 d.p.i. compared to the uninfected group. The expression pattern of IL-10 was significantly higher at 3 d.p.i. in the infected group of chickens compared to the uninfected group (P<0.05). The expression of IL-8 and IL-18 was not significantly different (P>0.05) between infected and uninfected groups.

**Figure 4:**
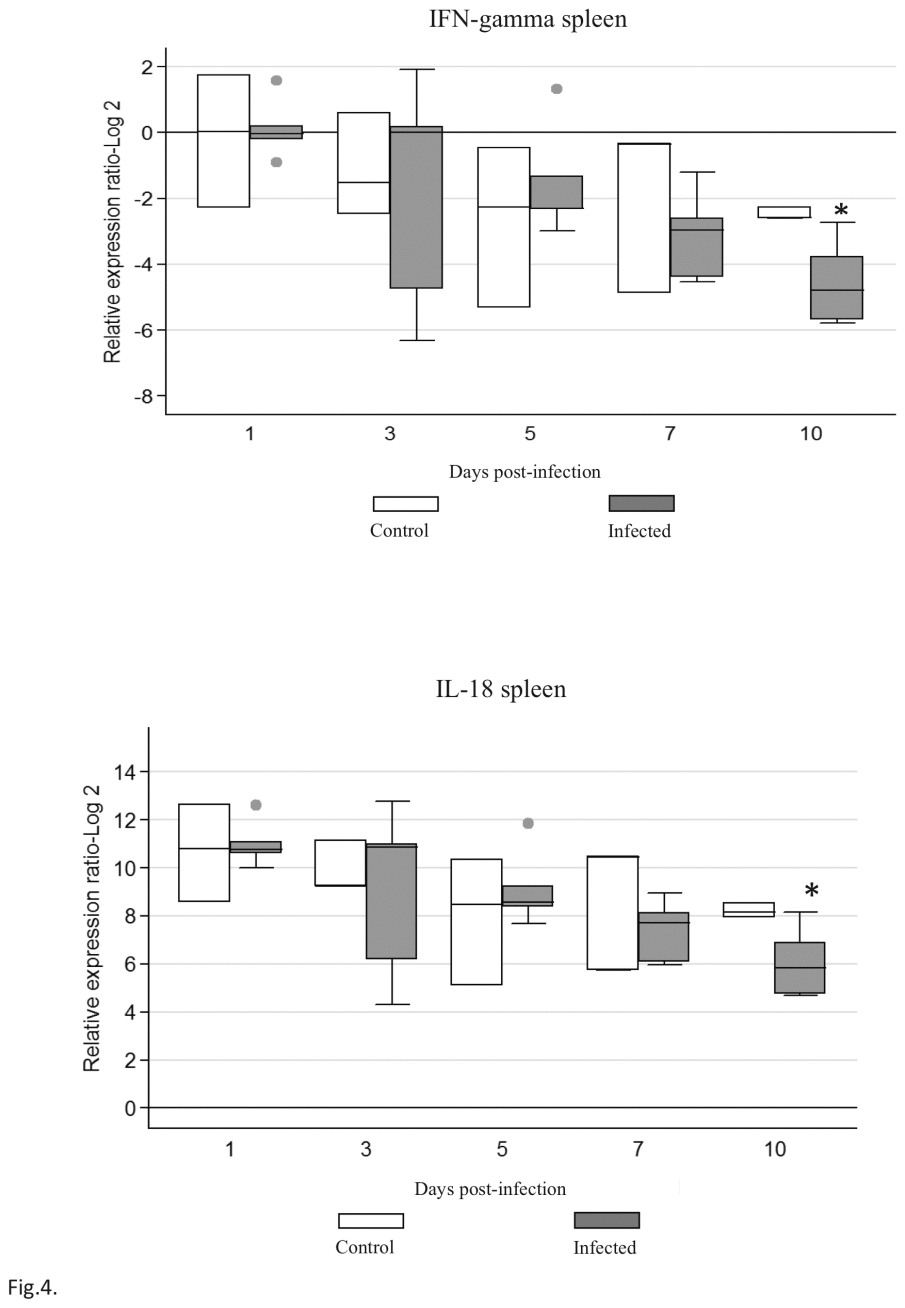
Cytokine mRNA expression in spleen of chickens infected with FAdV-4. The groups were: Uninfected (negative control), and FAdV-4 infected chickens using im inoculation. Target and reference gene expression was quantified by real-time RT-PCR using SYBR Green. Target gene expression is presented relative to β-actin expression and normalized to a calibrator. The results are diagrammed as whisker boxes with medians. Boxes represent interquartile ranges and whiskers indicate extreme values. The difference in cytokine expression between groups was assessed by Mann-Whitney test and comparisons were considered significant at P≤0.05 (*).● symbols represent values which were identified as outliers.

 In the spleen, both IFN-γ and IL-18 mRNA were detected at all-time-points (Figure 4). A statistically significant (*P*<0.05) down-regulation of both IFN-γ and IL-18 in the spleen was noted in the infected group at 10 d.p.i. 

## Discussion

There were no clinical signs, or gross and histopathological lesions in any of the inoculated birds by either inoculation route, even though they were inoculated with a high virus dose, suggesting it could be considered non-pathogenic. 

The lack of pathological changes was not due to lack of virus replication, since virus was found in tissues and cloacal swabs of the FAdV-4 infected birds; although in numerically lower quantities than previously reported for FAdV-8 and FAdV-9 [16,11]. In both the orally and im inoculated groups, the highest numbers of viral genome copies were detected in the cecal tonsil, in agreement with our work on FAdV-8 [16]. Ojkic and Nagy [29] reported that replication and distribution of another serotype virus (FAdV-9) in tissues are influenced by the route of inoculation. Since FAdV-4 is transmitted horizontally by the oral-fecal route [9], the im and the natural oral routes of infection were compared. In general, the orally inoculated group had a higher number of viral genome copies in contrast to our work on FAdV-8 [16], where the im-inoculated chicks had a higher number of viral genome copies. The reason for this difference might be due to the different serotypes used in these two studies. FAdV-4 ON1 was isolated from cloacal swabs until the end of the study (28 d.p.i.) in the im-inoculated group, while for the oral group, shedding was recorded only up to 21 d.p.i. Orally inoculated chickens had a significantly higher virus titer in feces than the im-inoculated group at all time points. Similar patterns of shedding up to 28 d.p.i. were also reported for FAdV-8 [16]. In further studies different breeds and sources of chickens should be considered in order to increase external validity of results.

 Ab response was significantly higher in FAdV-4 im-inoculated chicks than in the orally inoculated chicks. FAdV-8 and FAdV-9 also induced higher antibody levels when the birds were infected via the im route [16,29]. Since sera using a heterologous FAdV serotype, FAdV-9, were positive with ELISA, and although the S/P ratios were lower, these results confirm already publish data that heterologous virus can be used as an antigen to detect adenovirus Abs against other serotypes [33,13]. Importantly, we demonstrated that FAdV-4 ON1 was immunogenic and induced neutralizing antibodies in chickens. Therefore this isolate could be used as a live vaccine virus.

 The most notable features related to the genome of the FAdV-4 ON1 are its size and the presence of two fibers. According to previous research [18], FAdV-4 ON1 with 45667 bp in length is the largest AdV genome reported to date. The two fiber genes located adjacent to one another in the central genomic region showed a similar β-strand arrangement to FAdV-1 fiber 1 and fiber 2 [19,12]. Authors speculated that the FAdV-4 fiber 2 might bind a receptor other than CAR and determine the tissue tropism of FAdV-4, possibly leading to the unique clinical features associated with infection with virulent FAdV-4 [18].

Cytokine gene expression associated with T helper (Th)1, pro-inflammatory, and regulatory activities, namely IFN-γ, IL-18, IL-10 and IL-8, was investigated in the liver, spleen and cecal tonsils, and gene activation at early times after infection was shown. The expression of cytokines has been studied following FAdV-8 infection [15], however, this is the first report on the cytokine repertoire associated with FAdV-4 infection. Enhanced expression of IFN-γ in the liver was noted, with a significant increase in expression at 3 d.p.i. IFN-γ is considered an essential cytokine in host defense against a variety of pathogens. For example IFN-γ inhibits Marek’s disease virus (MDV) replication by inducing nitric oxide synthesis [41], reduces replication of coccidian parasites [24], and inhibits Rous sarcoma virus *src* oncogene-induced tumor growth [34]. IFN-γ also inhibits transgene expression from adenoviral vectors *in vitro* by a transcription-related mechanism [39].

The IL-10 in the liver was significantly up-regulated at 3 d.p.i. in the infected group, when compared to that of the negative control birds. Our finding that IL-10 was up-regulated following FAdV-4 infection is in agreement with a previous report [15], and may indicate an immune evasive mechanism used by the virus to skew the immune response, supporting persistence of the virus [17]. IL-10 has both immunosuppressive and immunostimulating effects on cells of the adaptive immune system [20]. Simultaneous expression of IFN-γ and IL-10 also has been reported after infection with porcine reproductive and respiratory syndrome virus [21]. More recently Parvizi et al. [30] reported persistent up-regulation of IFN-γ and IL-10 in a MDV-susceptible line of chickens. In humans, up-regulation of both IFN-γ and IL-10 is directly related to IL-12 stimulation of CD4^+^ and CD8^+^ T cells [14]. 

 In summary, the lack of clinical signs, and gross and histopathological lesions indicated that FAdV-4 ON1 was a non-pathogenic virus. However, virus replication was demonstrated in tissues and it induced a strong Ab response. The prolonged virus shedding could be advantageous in vaccinated poultry flocks, ensuring a long and uniform exposure to the virus. Infection with this virus was associated with increased expression of IFN-γ and IL-10 in the liver and decreased expression of IFN-γ and IL-18 in the spleen, providing useful information about important mediators involved in immunity against virus infection. Our study demonstrated that the FAdV-4 ON1 potentially could be used as a live vaccine against HHS and developed as vaccine vector. However, further research is needed to confirm protective nature of this vaccine candidate. 

## References

[1] Abdul-Careem MF, Hunter BD, Parvizi P, Haghighi HR, Thanthrige-Don N et al. (2007) Cytokine gene expression patterns associated with immunization against Marek’s disease in chickens. Vaccine 25: 424-432. doi:10.1016/j.vaccine.2006.08.006. PubMed: 17070626. 17070626

[2] Abdul-Careem MF, Hunter BD, Sarson AJ, Mayameei A, Zhou H et al. (2006) Marek’s disease virus-induced transient paralysis is associated with cytokine gene expression in the nervous system. Viral Immunol 19: 167-176. doi:10.1089/vim.2006.19.167. PubMed: 16817759. 16817759

[3] Abe T, Nakamura K, Tojo H, Mase H, Shibahara T et al. (1998) Histology, immunohistochemistry, and ultrastructure of hydropericardium syndrome in adult broiler breeders and broiler chicks. Avian Dis 42: 606-612. doi:10.2307/1592690. PubMed: 9777164. 9777164

[4] Adair BM, Fitzgerald SD (2008) Adenovirus infections. In: Saif YM, Fadly AM, Glisson JR, McDougald LR, Nolan LK, Swayne DE editors. Disease of poultry. Ames: Blackwell Publishing Professional pp. 251-291.

[5] Alexander HS, Huber P, Cao JX, Krell PJ, Nagy É (1998) Growth characteristics of fowl adenovirus type 8 in a chicken hepatoma cell line. J Virol Methods 74: 9-14. doi:10.1016/S0166-0934(98)00062-7. PubMed: 9763123. 9763123

[6] Anjum AD (1990) Experimental transmission of hydropericardium syndrome and protection against it in commercial broiler chickens. Avian Pathol 19: 655-660. doi:10.1080/03079459008418721. PubMed: 18679979. 18679979

[7] Corredor JC, Nagy É (2010) A region at the left end of the fowl adenovirus 9 genome that is non-essential in vitro has consequences in vivo. J Gen Virol 91: 51-58. doi:10.1099/vir.0.013839-0. PubMed: 19759237. 19759237

[8] Corredor JC, Nagy É (2010) The non-essential left end region of the fowl adenovirus. p. 9. 10.1016/j.virusres.2010.01.014 20132849

[9] Cowen B (1992) Inclusion body hepatitis-anaemia and hydropericardium syndrome: aetiology and control. Worlds Poult Sci J 48: 247-253. doi:10.1079/WPS19920019.

[10] Cheema AH, Ahmad J, Afzal A (1989) An adenovirus infection of poultry in Pakistan. Rev Sci Tech Off Int Epiz 8: 789-795. 10.20506/rst.8.3.420 32344955

[11] Deng L, Sharif S, Nagy E (2013) Oral inoculation of chickens with a candidate fowl adenovirus 9 vector. Clin Vaccine Immunol. doi:10.1128/CVI 00187-13 PMC3754522 23740924

[12] El Bakkouri M, Seiradake E, Cusack S, Ruigrok RWH, Schoehn G (2008) Structure of the C-terminal domain of the fowl adenovirus type 1 short fibre. Virology 378: 169-176. doi:10.1016/j.virol.2008.05.011. PubMed: 18561970. 18561970

[13] Erny K, Pallister J, Sheppard M (1995) Immunological and molecular comparison of fowl adenovirus serotypes 4 and 10. Arch Virol 140: 491-501. doi:10.1007/BF01718426. PubMed: 7733822. 7733822

[14] Gerosa F, Paganin C, Peritt D, Paiola F, Scupoli MT et al. (1996). Interleukine-12 primes human CD4 and CD8 T cell clones for high production of both interferon-gamma and interleukine-10. J Exp Med 183:2559-69. 8676077 10.1084/jem.183.6.2559 PMC2192598

[15] Grgić H, Sharif S, Haghighi HR, Nagy É (2013) Cytokine patterns associated with a serotype 8 fowl adenovirus infection. Viral Immunol 26: 143-149. doi:10.1089/vim.2012.0078. PubMed: 23537431. 23537431

[16] Grgić H, Yang D-H, Nagy É (2011) Pathogenicity and complete genome sequence of fowl adenovirus serotype 8 isolate. Virus Res 156: 91-97. doi:10.1016/j.virusres.2011.01.002. PubMed: 21237223. 21237223

[17] Grgić H, Philippe C, Ojkić D, Nagy É (2006) Study of vertical transmission of fowl adenoviruses. Can J Vet Res 70: 230-233. PubMed: 16850947. 16850947 PMC1477927

[18] Griffin BD, Nagy É (2011) Coding potential and transcript analysis of fowl adenovirus 4: insight into upstream ORFs as common sequence features in adenoviral transcripts. J Gen Virol 92: 1260-1272. 21430092 10.1099/vir.0.030064-0

[19] Guardado-Calvo P, Llamas-Saiz AL, Fox GC, Langlois P, van Raaij MJ (2007) Structure of the C-terminal head domain of the fowl adenovirus type 1 long fiber. J Gen Virol 88: 2407- 2416. doi:10.1099/vir.0.82845-0. PubMed: 17698649. 17698649

[20] Hess M, Raue R, Prusas C (1999) Epidemiological studies on fowl adenoviruses isolated from cases of infectious hydropericardium. Avian Pathol 28: 433-439. doi:10.1080/03079459994443. 26911596

[21] Johnsen CK, Bøtner A, Kamstrup S, Lind P, Nielsen J (2002) Cytokine mRNA profiles in bronchoalveolar cells of piglets experimentally infected in utero with porcine reproductive and respiratory syndrome virus: association of sustained expression of IFN-gamma and IL- 10 after viral clearance. Viral Immunol 15: 549-556. doi:10.1089/088282402320914494. PubMed: 12513926. 12513926

[22] Johnson MA, Pooley C, Ignjatovic J, Tyack SG (2003) A recombinant fowl adenovirus expressing the S1 gene of infectious bronchitis virus protects against challenge with infections bronchitis virus. Vaccine 21: 2730-2736. doi:10.1016/S0264-410X(03)00227-5. PubMed: 12798610. 12798610

[23] Khawaja DA, Ahmad S, Rauf MA, Zulfiqar MZ, Mahmood SMI et al. (1988) Isolation of an adenovirus from hydropericardium syndrome in broiler chicks. Pak J Vet Res 1: 2-17.

[24] Lillehoj HS, Choi KD (1998) Recombinant chicken interferon-gamma-mediated inhibition of *Eimeria* *tenella* development *in* *vitro* and reduction of oocyst production and body weight loss following *Eimeria* *Acervulina* challenge infection. Avian Dis 42: 307-314. doi:10.2307/1592481. PubMed: 9645322. 9645322

[25] Mansoor MK, Hussain I, Arshad M, Muhammad G (2011) Preparation and evaluation of chicken embryo-adapted fowl adenovirus serotype 4 vaccine in broiler chickens. Trop Anim Health Prod 43: 331-338. doi:10.1007/s11250-010-9694-z. 20878234

[26] Mazaheri A, Prusas C, Vo M, Hess M (1998) Some strains of serotype 4 fowl adenovirus cause inclusion body hepatitis and hydropericardium syndrome in chickens.Avian Pathol 27: 269-276. doi:10.1080/03079459808419335. PubMed: 18483997. 18483997

[27] Nakamura K, Mase M, Yamaguchi S, Shibahara T, Yuasa N (1999) Pathologic study of specific pathogen free chicks and hens inoculated with adenovirus isolated from hydropericardium syndrome. Avian Dis 43: 414-423. doi:10.2307/1592638. PubMed: 10494409. 10494409

[28] Ojkic D, Nagy É (2001) The long repeat region is dispensable for fowl adenovirus replication in vitro. Virology 283: 197-206. doi:10.1006/viro.2000.0890. PubMed: 11336545. 11336545

[29] Ojkic D, Nagy É (2003) Antibody response and virus tissue distribution in chickens inoculated with wild-type and recombinant fowl adenoviruses. Vaccine 22: 42-48. doi:10.1016/S0264-410X(03)00544-9. PubMed: 14604569. 14604569

[30] Parvizi P, Read LR, Abdul-Careem MF, Sarson AJ, Lusty C et al. (2009) Cytokine gene expression in splenic CD4^+^ and CD8^+^ T cell subsets of genetically resistant and susceptible chickens infected with Marek’s disease virus. Vet Immunol Immunopathol 132: 209-217. doi:10.1016/j.vetimm.2009.06.009. PubMed: 19615758. 19615758

[31] Pfaffl MW (2001) A new mathematical model for relative quantification in real-time RT- PCR. Nucleic Acids Res 29: 2003-2007. doi:10.1093/nar/29.10.2003. PubMed: 11353068. 11328886 PMC55695

[32] Philippe C, Grgic H, Nagy É (2005) Inclusion body hepatitis in young broiler breeders associated with a serotype 2 adenovirus in Ontario, Canada. J Appl Poult Res 14: 588-593.

[33] Philippe C, Grgić H, Ojkić D, Nagy É (2007) Serologic monitoring of a broiler breeder flock previously effected by inclusion body hepatitis and testing the progeny for vertical transmission of fowl adenoviruses. Can J Vet Res 71: 98-102. PubMed: 17479772. 17479772 PMC1829188

[34] Plachý J, Weining KC, Kremmer E, Puehler F, Hala K et al. (1999) Protective effects of type I and type II interferons toward Rous sarcoma virus-induced tumors in chickens. Virology 256: 85-91. doi:10.1006/viro.1999.9602. PubMed: 10087229. 10087229

[35] Rasmussen R (2001) Quantification on the LightCycler. In Meuer S, Wittwer C, Nakagawara K, editors. Rapid Cycle Real-time PCR, Methods and Application. Heidelberg: Springer Verlag Press . pp. 21-34

[36] Saifuddin M, Wilks CR (1991) Pathogenesis of an acute viral hepatitis: inclusion body hepatitis in the chicken. Arch Virol 116: 33-43. doi:10.1007/BF01319229. PubMed: 1848068. 1848068

[37] Schonewille E, Jaspers R, Guntram P, Hess M (2010) Specific-pathogen-free chickens vaccinated with a live FAdV-4 vaccine are fully protected against a severe challenge even in the absence of neutralizing antibodies. Avian Dis 54: 905-910. doi:10.1637/8999-072309-Reg.1. PubMed: 20608537. 20608537

[38] Sheppard M, Werner W, Tsatas E, McCoy R, Prowse S et al. (1998) Fowl adenovirus recombinant expressing VP2 of infectious bursal disease virus induces protective immunity against bursal disease. Arch Virol 143: 915-930. doi:10.1007/s007050050342. PubMed: 9645198. 9645198 PMC7087160

[39] Sung RS, Qin L, Bromberg JS (2001) TNF alpha and IFN gamma induced by innate anti- adenoviral immune responses inhibit adenovirus-mediated transgene expression. Mol Ther 3: 757-767. doi:10.1006/mthe.2001.0318. PubMed: 11356080. 11356080

[40] Toro H, Prusas R, Raue R, Cerda L, Geisse C et al. (1999) Characterization of fowl adenoviruses from outbreaks of inclusion body hepatitis hydropericardium syndrome in Chile. Avian Dis 43: 262-270. doi:10.2307/1592616. PubMed: 10396639. 10396639

[41] Xing Z, Schat KA (2000) Inhibitory effects on nitric oxide and γ interferon on *in* *vitro* and *in* *vivo* replication of Marek’s disease virus. J Virol 74: 3605-3612. doi:10.1128/JVI.74.8.3605-3612.2000. PubMed: 10729136. 10729136 PMC111870

